# Medical-Grade Honey Enhances the Healing of Caesarean Section Wounds and Is Similarly Effective to Antibiotics Combined with Povidone-Iodine in the Prevention of Infections—A Prospective Cohort Study

**DOI:** 10.3390/antibiotics12010092

**Published:** 2023-01-05

**Authors:** Amadou Bocoum, Senna J. J. M. van Riel, Soumana Oumar Traoré, Elisabeth Florine Ngo Oum II, Youssouf Traoré, Augustin Tioukani Thera, Seydou Fané, Bakary Tientigui Dembele, Niels A. J. Cremers

**Affiliations:** 1 Gynecology-Obstetrics Department, Faculty of Medicine and Odonto-Stomatology, Center Hospitalier Universitaire (CHU) Gabriel Touré, Bamako, Mali; 2 Department of Obstetrics and Gynecology, Zuyderland Medical Center, 6162 BG Sittard-Geleen, The Netherlands; 3 Department of Gynecology and Obstetrics, Maastricht University Medical Center, 6202 AZ Maastricht, The Netherlands; 4 Department of General Surgery, Faculty of Medicine and Odonto-Stomatology, CHU Gabriel Touré, Bamako, Mali; 5 Triticum Exploitatie BV, 6222 NK Maastricht, The Netherlands

**Keywords:** caesarean section, medical-grade honey, postoperative wounds, surgical site infections, wound care, antibiotics, antibiotic resistance, suture techniques

## Abstract

Caesarean sections (CS) are becoming increasingly popular. The antibiotic resistance crisis and relentless risk of infections, especially in developing countries, demand alternative treatment options. Medical-grade honey (MGH) exerts antimicrobial and healing properties. This study aims to evaluate the effect of MGH treatment on CS wound healing and postoperative complications when compared to conventional treatment (antibiotics in combination with povidone-iodine). In this prospective cohort study, 766 CS patients were included and evenly divided into two groups. The treatment group (n = 383) received an MGH-based formulation (L-Mesitran Soft) and the control group (n = 383) received antibiotics (Amoxicillin) combined with povidone-iodine. The wound healing time and complication rate were determined for both groups, and subsequently, predisposing factors for complications among the baseline characteristics and non-patient-related parameters were determined. The baseline characteristics were similar for both study groups, supporting a homogenous distribution. Postoperative complications were experienced by 19.3% of the patients in the control group and 18.8% in the treatment (MGH) group. The treatment group experienced significantly more superficial pus discharge than the control group, while the latter experienced significantly more deeper pus discharge. BMI, age, duration of hospitalization, anesthesia, and duration of CS could affect the complication risk. MGH significantly enhanced wound healing until day 42. On average, the healing time with MGH was 19.12 ± 7.760 days versus 24.54 ± 8.168 days in the control group. MGH is a potent alternative treatment to antibiotics and povidone-iodine because while the complication risk is similar, MGH has additional benefits. MGH promotes wound healing and does not bear the risk of resistance.

## 1. Introduction

More than half a million women die from pregnancy-related causes every year, of which 99% are in low- and middle-income countries [1]. A caesarean section (CS) is a surgical procedure that has been practiced since at least the eighth century BC, typically when the mother was dying or dead, in an attempt to rescue the fetus [2]. The discovery of anesthesia in the 19th century and optimization of surgical and aseptic techniques, and the introduction of blood banks and antibiotics in the 20th century, led to an improved outcome of CSs [2,3]. This subsequently led to a strong increase in CSs being performed. It is estimated that currently 21.1% of women globally give birth using CS and this number is expected to increase to 28.5% by 2030 [4]. Interestingly, there is large variability across different regions. For example, 42.8% of the women in Latin America and the Caribbean, 31.6% in Northern America, 25.7% in Europe, 23.1% in Asia, and 9.2 % in Africa (of which 5.0% are in Sub-Saharan Africa) give birth via CS [4]. In developed countries, CSs may be performed without medical indication (overuse), not leading to clinical benefits and only burdening patients and the health care system [4,5,6]. In developing countries, CSs are underused due to inadequate access to emergency obstetric care, or because the procedure is too risky [4,5,6]. As a consequence, maternal and perinatal mortality rates after CS remain unacceptably high, especially in developing countries.

The average maternal mortality rate is 0.76% (7.6 per 1000 CSs), with women from Sub-Saharan Africa at the highest risk of death (10.9 per 1000 CSs) and women from Europe and Central Asia at the lowest risk (0.3 per 1000 CSs) [7]. The perinatal mortality rate is 8.47% (84.7 per 1000 CSs), with the highest rate in the Middle East and North Africa (354.6 per 1000 CSs) followed by Sub-Saharan Africa (100.4 per 1000) and the lowest risk in Europe and Central Asia (1.8 per 1000 CSs). These data also show that there is a big difference between developed and developing countries [7].

Causes of maternal mortality include post-partum hemorrhage, sepsis (surgical site infection (SSI)), pre-eclampsia, venous thromboembolism, and anesthesia-related complications [6,8]. These complications contribute not only to mortality, but also to morbidity in patients [8]. Surgical site infections (SSIs) are defined as infections occurring within 30 days post-operation and affecting superficial or deep incisional sites, or deeper-lying spaces or organs [9,10]. SSIs are also associated with increased healthcare costs, longer hospitalization, and more patient dissatisfaction [11]. Globally, the rate of SSI following CSs varies roughly between 3 to 15%, with higher levels in developing countries [12]. However, higher SSI rates are also described and attributed to the quality of care, study sample size, and predisposing factors (e.g., diabetes mellitus, obesity, smoking, malnutrition, and age) [13,14,15,16]. It is estimated that half of all SSIs can be prevented [11,13], as was recently proven in Dublin, Ireland [12].

SSIs can be prevented via preoperative, intraoperative, and postoperative measures. Since SSIs predominantly occur superficially and are less often observed deeper in the incisional site and deeper-lying space or organs [13,17], optimizing postoperative wound care may be an important step in strongly reducing SSIs. Currently, there are no clear guidelines for wound care following CS, and treatment depends on local hospital protocols and the experience of the clinicians [17,18]. Usually, a plaster or bandage will be applied with or without some kind of topical treatment, such as povidone-iodine, Vaseline, or Bepanthen cream. Moreover, an antibiotic cream may be applied locally, or systemic antibiotics can be administered. The high level of SSIs and the rise in antimicrobial resistance towards antibiotics demand alternative or complementary treatments.

Medical-grade honey (MGH) may be a potent alternative treatment option, because of its antimicrobial and wound healing activities [19]. Honey has roots as a treatment option for wounds and local infections in antiquity [20,21]. MGH adheres to strict criteria to guarantee its safety and efficacy for wound care [22]. It has multiple antimicrobial mechanisms, including acidic pH, osmotic activity, the slow release of hydrogen peroxide, and the presence of antimicrobial molecules [23,24,25]. This makes MGH effective against a broad range of micro-organisms, without the risk of developing resistance to honey [26]. Besides acting as an agent against established infections, there is evidence of MGH’s ability to combat infections prophylactically [27,28,29]. In addition, MGH also exerts wound healing properties through a myriad of mechanisms. Specifically, MGH creates a moist wound environment, promotes (autolytic) debridement, stimulates angiogenesis and re-epithelialization, and optimizes wound healing through its anti-inflammatory, anti-oxidative, and immunomodulatory effects [25,30,31]. MGH can be used in a large number of applications, of which CS is just one possible application. MGH is widely accessible, and easy to apply and follow for a reasonable period.

These combined properties of MGH present an exciting opportunity to investigate its clinical use during postoperative CS wound care. This study aims to investigate whether topical treatment with MGH is effective in preventing infections and enhances wound healing after CS when compared to conventional treatment (systemic antibiotics and topical povidone-iodine).

## 2. Results

### 2.1. MGH Is as Effective as Antibiotics Combined with Povidone-Iodine in Controlling Postoperative Complications and Infections

The baseline characteristics were comparable between the two study groups (Table 1). Patients were treated with either MGH (treatment group) or with conventional treatment consisting of antibiotics in combination with povidone-iodine (control group). The risks of complications and infections were determined per group (Table 2). The requirement to be classified as having a postoperative complication is having pain at the incision site and having another complaint. The rate of postoperative complications in the control (antibiotics combined with povidone-iodine) and treatment (MGH) groups were 19.3% and 18.8%, respectively. There was no significant difference between the study groups (relative risk (RR) of 0.9730 with a 95% confidence interval (CI) of 0.7267–1.3028 and a *p*-value of 0.8540). SSIs were considered to be present when patients experienced pain and there was pus discharge from the wounds. The SSI rates in the control and treatment groups were 15.9% and 14.4%, respectively. Again, there was no significant difference between the study groups (RR: 0.9016, CI: 0.6445–1.2615, *p*-value: 0.5456). Thus, the risk of developing postoperative complications or infections was the same in both groups.

For the rest of the measured outcomes, sub-analyses were performed in which the subgroups were based on the presence or absence of postoperative complications.

### 2.2. The Type of Complication Varied between the Study Groups

Information was collected about the type of complication per individual patient. The distribution of these different types of complications is presented per group (Table 3). Since pain at the surgical site was a prerequisite to classifying it as a postoperative complication, all patients within this analyzed subgroup experienced pain. Hence, similar to the risk of complications, there was no difference regarding the prevalence of pain between the two study groups (RR: 0.9730, CI: 0.7267–1.3028, *p*-value: 0.8540).

Within each group, there may be an increased risk of a certain type of complication (Table 3). In both groups, the majority of the complications were superficial pus discharge. The risk of this type of complication was significantly higher (3.88-fold and 6.30-fold for the control and treatment groups, respectively) when compared to the reference complication. In the control group, there was also a significantly increased risk of having deep pus discharge. When comparing the types of complications between the two study groups, a significant 4.9-fold increased risk (RR = 0.21) of deep pus discharge was found for the control group (40.5% vs. 8.3%). In the treatment group, a significant 1.62-fold increase in superficial pus discharge (68.1% vs. 41.9%) and a significant 2.67-fold increase in wound bleeding (18.1% vs. 6.8%) were found when compared to the control group. Other types of complications were less common and not significantly different, but could be more related to the study groups. For example, elevated levels of crust formation in the control group may be attributed to the use of povidone-iodine, which can dry out wounds, while in the treatment group wound exudate may be higher as a result of the osmotic activity of the MGH.

Another complication that could be present, but does not, by definition, need to be related to the CS, is fever. The number of patients per (sub)group who developed a fever within the first 30 days after the CS procedure is presented in Table 4. The number of patients within the control group who developed a fever after their CS was significantly (10.44-fold) higher in the subgroup with patients who had complications (5 out of 69) than in those without complications (2 out of 302), while there was no significant difference in the treatment group (1 out of 71 in the case of complications vs. 2 out of 309 when there was no complication). When comparing the limited number of patients with fever within the subgroups with complications, there was no significant difference in the control group when compared to the treatment group (RR: 0.2056, CI: 0.0246–1.7167, *p*-value: 0.144).

Thus, the type of complication was demonstrated to be study group dependent. However, there may also be other internal and external factors that could have influenced the risk of complications.

### 2.3. Several Predisposing Factors Increase the Risk of Complications after a CS

Next, we evaluated whether any of the baseline characteristics or other factors contribute to a higher chance of complications. The baseline characteristics are already shown in Table 1. No significant differences in prevalence between the groups existed for the included baseline variables, and the two groups were homogenously distributed (Table 1). In Table 5, the influence of patient-related baseline characteristics on the complication rate is presented.

The majority of patients are between 20 and 34 years of age for both groups, and therefore, this age group of the control group was selected as a reference. Patients of 19 years and younger within the treatment group had a 2.17-fold significantly decreased risk (RR: 0.47, CI: 0.23–0.95) of developing complications. No other significant differences were found regarding age. The number of pregnancies per patient was similar within the study groups, as was the number of children born per patient. Patients in the control group had a significantly (3.15-fold (CI: 1.44–6.90) and 2.63-fold (CI: 1.11–6.22)) higher risk when they were underweight (≤18.5) or morbidly obese (>40), respectively. No other significant differences within the study groups were found for the BMI, education level, or occupation of the patients. When comparing the prevalence of complications for these baseline characteristics between the groups (Table 5), there were no significant differences found. Thus, we only found some predisposing factors that were unrelated to the study groups.

Additionally, some patient-independent factors were evaluated as possible predisposing factors to complications (Table 6). There was no increased risk of complications depending on the mode of admission to the hospital (own initiative or upon referral). Within the control group, a hospitalization duration of 5–10 days was associated with a significant 1.89-fold decreased risk of complications (RR: 0.53, CI: 0.29–0.96), while hospitalization of 10–15 days was associated with a 2.63-fold (CI: 1.41–4.93) increased risk of complications. The presence of membranes during CS or the delay between the rupture of the membranes and the CS procedure were not associated with an increased risk of complications in any of the two study groups. There were significantly more complications found after general anesthesia than with spinal or epidural anesthesia, irrespective of the study group (1.78-fold and 1.65-fold for the control and treatment groups, respectively). A duration of 45–60 min for the CS procedure was associated with a 1.94-fold (CI: 1.20–3.15) increased risk of complications for the treatment group. There were no other significant predisposing factors found within the study groups. When comparing the risk of complications for these parameters between the groups (Table 6), the only observed significant difference was a hospitalization duration of 5–10 days. This risk was 2.12 times (CI: 1.10–4.09) higher in the treatment group when compared to the control group; however, this may also be related to the reduced risk within the control group.

Another important aspect of CS treatment besides the risk of complications is wound healing time.

### 2.4. The Application of MGH for Postoperative Wound Care Leads to Faster Wound Healing

**Figure 1:**
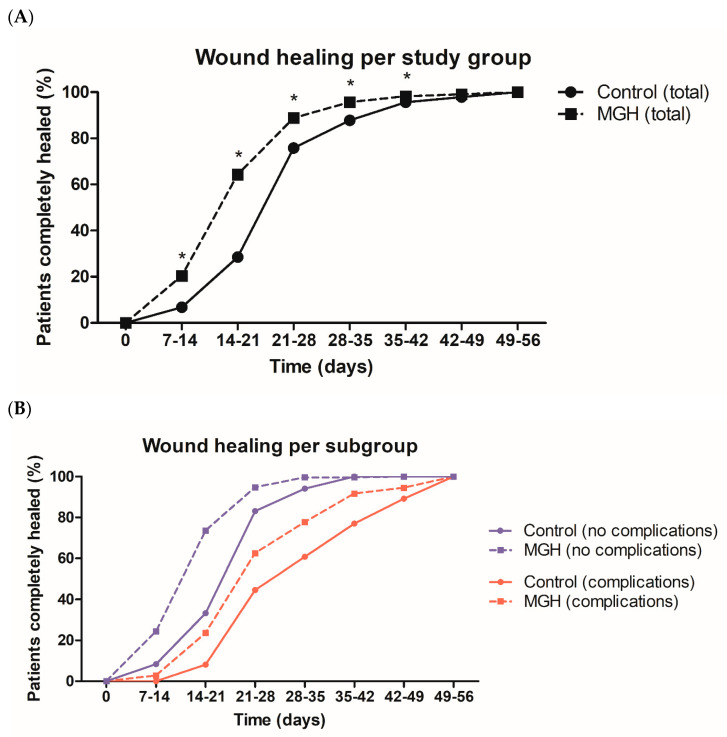
(**A**) Wound healing progression per study group. (**B**) Wound healing progression per subgroup (study groups divided into groups with and without complications). The control (sub)groups (antibiotics + povidone-iodine) are presented by solid lines and circles, and the treatment (sub)groups (MGH) are presented by dashed lines with squares. Purple lines represent the subgroup without complications and red lines the subgroups with complications). An asterisk (*) above the line represents a significant difference between the groups at that certain time point.

Wound healing time was classified as the time in days taken for the CS wound to completely close. An overview of the wound healing progression per study group is presented in Table 7 and Figure 1A. In the control group (antibiotics + povidone-iodine), the majority of wounds healed before 28 days, with an average of 24.54 ± 8.168 days (minimum of 11 and maximum of 56 days). In the treatment group (MGH), most of the wounds were healed before 21 days, with an average of 19.12 ± 7.760 days (minimum of 11 and maximum of 56 days). Healing time was significantly enhanced in the treatment group from the first measured time point (day 7) until day 42 when compared to the control group (Figure 1A). After this time point, a small number of patients who had wounds remained (17 in the control group and 7 in the treatment group). The risk of developing postoperative complications increased with healing time in both groups (Table 7). This was significantly different from day 28 onwards in the control group and day 21 onwards in the treatment group. This is clearly visible when the subgroups with and without complications are presented separately per study group (Figure 1B). There was a shift in the number of patients who had completely healed CS wounds in both study groups when there was no complication when compared to the subgroups with complications.

**Figure 2:**
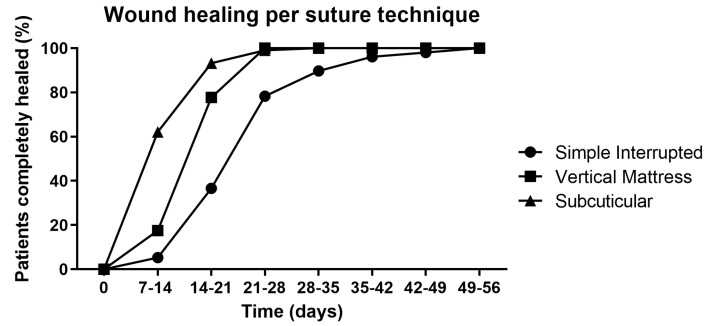
Wound healing progression per suture technique. The two study groups are combined because there was no significant association between the study group and the risk of complications.

Since there was no significant association between the suture technique and complication risk within and between the study groups (Table 6), we next investigated if the suture technique may have affected the healing time (Table 8). The simple interrupted suture technique was the most often used in both groups. In line with the healing time of the two study groups, the treatment group healed faster than the control group, irrespective of the suture technique. This can be observed by the change in the relative distribution of completely healed patients between the two study groups. For the first time points, the majority of patients for a certain suture technique healed in the treatment group (days 7–21), and at later time points (days 21–56), the control group was larger. The healing times of the two study groups per suture technique are combined in Figure 2 to obtain a general impression of the healing time per suture technique. It can be observed that the subcuticular suture technique heals the fastest, followed by the vertical mattress suture technique, and then, the simple interrupted suture technique. However, the latter technique is used in 81.5% of all cases, and thus, there may be underlying reasons or preferences for using this or the other two suture techniques; subsequently, this may potentially have affected the healing time.

## 3. Discussion

In this prospective cohort study, 766 patients were included and evenly distributed into two equal study groups of 383 patients, receiving either antibiotics in combination with povidone-iodine (control group) or MGH (treatment group). The baseline characteristics were similar for both study groups, supporting a homogenous distribution of the patients. Postoperative complications were experienced by 19.3% of the patients in the control group and 18.8% in the treatment (MGH) group, with 15.9% and 14.4% having SSI, respectively. There was no significant difference in the complication and infection rates between the two study groups. Several baseline characteristics and other non-patient-related factors affected the risk of complications. The presence of complications delayed wound healing in both study groups. Furthermore, MGH strongly enhanced wound healing speed when compared to conventional treatment (control).

Since there was no difference in the complication and infection rates, it can be stated that the antimicrobial activity of MGH is as potent as that of conventional antibiotics in combination with the antiseptic agent povidone-iodine. Moreover, the used MGH formulation (L-Mesitran Soft) has previously demonstrated strong prophylactic activity [27,28]. An added benefit of MGH is that there is no risk of bacteria developing resistance, and this would help to reduce the antimicrobial resistance crisis [26,32]. In developing countries, there is a high risk of antimicrobial resistance, due to the misuse of antimicrobials, over-the-counter availability, non-compliance, and unregulated supply chains [33]. To illustrate this, a previous study in Rwanda found that 84.6–100% of the infected CS cases were resistant to commonly used antibiotics (ampicillin, ceftriaxone, and cefepime) [34]. Infections arose despite 88.8% of the cohort receiving pre-operative antibiotics (94% ampicillin) and 95.9% receiving postoperative antibiotics (52.3% ampicillin, 48.1% gentamicin, 46.3% metronidazole, and 41.3% ceftriaxone) [34]. Another advantage of MGH over antibiotics is that MGH will not end up in the circulation of the patient, and subsequently, in the milk of breastfeeding women. Antibiotics will likely always end up in the milk (from roughly 0.05% to 10.6%), depending on the antibiotic, its hydrophilicity and concentration, and different factors such as absorption, distribution, metabolism, and elution [35]. Even low doses of antibiotics in the milk can harm the infant by changing the microbiome, increasing antibiotic-resistant genes and the risk of allergies, and contributing to morbidities such as disturbances in brain development, immunity, and behavior, and obesity [35,36].

Interestingly, there was a significant difference in the types of complications per study group. Patients within the treatment group experienced more superficial pus discharge and wound bleeding; however, both symptoms may be related to the nature of MGH. MGH creates a moist wound environment and its osmotic activity attracts lymph fluid to the wound bed [31]. The control group had significantly more patients with deep pus discharge and fever. These findings suggest that the complications in the control group were more severe than in the treatment group. Several baseline characteristics affected the risk of complications, e.g., patients in the control group who were underweight or had morbid obesity had an increased risk, and patients in the treatment group who were younger than 19 years of age had a decreased risk. Aging and extreme BMIs have previously been associated with an increased risk of SSIs [37,38,39,40,41,42,43].

Other factors that could influence the risk of complications were the type of anesthesia, and the duration of hospitalization and CS surgery. In both groups, general anesthesia was associated with an increased risk. This can be explained by the fact that emergency and more complicated operations are more likely to require general anesthesia and more often result in a bad outcome. In the control group, long hospitalization (10–15 days) was associated with an increased risk, while hospitalization of 5–10 days reduced the risk of complications. As a consequence of the reduced risk after 5–10 days of hospitalization in the control group, the risk in the treatment group was higher when comparing the two study groups. These findings are in line with previous studies demonstrating that general anesthesia, and longer hospitalization and CS procedure duration are associated with higher risks of infection [38,44,45,46].

One of the most interesting findings of this study is that MGH significantly enhanced wound healing time from day 7 until day 42. On average, the healing time of the control group was 24.54 ± 8.168 days, while the healing of the treatment group was 19.12 ± 7.760 days.

MGH has antimicrobial activity and promotes healing via multiple mechanisms [20,21,22]. The antiseptic povidone-iodine may exert antimicrobial activity, but does not show wound healing-promoting properties [47]. In a recent systematic review and meta-analysis, twelve studies with a total of 1236 patients were included to compare the efficacy of honey and povidone-iodine. Honey reduced healing time, hospitalization, and pain when compared to povidone-iodine treatment [48]. The suture technique did not influence the complication rate but could influence the duration of wound healing. Wounds sutured with the vertical mattress suture or subcuticular technique healed faster than those wounds sutured with the simple interrupted suture technique.

The positive results of our study are in line with other studies. Several other studies also investigated the efficacy of honey for the treatment of CS. In a study on 53 patients with CS infections, topical honey treatment (n = 27) led to faster wound healing and bacterial resolution, decreased hospitalization, and reduced re-suturing, when compared to local antiseptics (povidone-iodine or 70% ethanol, n = 26) [49]. In a triple-blind randomized controlled trial with 129 CS patients (37 honey, 38 placebo, and 54 control), twice daily topical application of honey for 14 days reduced pain on days 7 and 14 and the need for analgesics compared to the placebo and control groups [50]. The same research group, in another publication with the same experimental setup, showed that honey decreased redness, edema, and hematoma when compared to the placebo; however, no data regarding the control group were presented [51]. In another double-blind RCT, 124 patients (44 honey, 40 placebo, and 40 control) with CS experienced no improvement in wound healing, pain level, or scar formation on days 10 or 40 post-surgery [52]. In an observational study on 776 patients, the application of honey after CS (n = 186) reduced the infection rate from 5.42% to 2.15% in comparison to the control (n = 590) [53].

The findings in this study are thus in line with previous beneficial findings. The broad-spectrum antimicrobial activity of MGH makes it effective in preventing infections, while also promoting wound healing. Previous studies have demonstrated that MGH is effective in resolving infections in several types of wounds caused by different types of bacteria, and can even eradicate biofilms [20,30,31,54]. The supplements in the MGH formulation L-Mesitran further enhance these beneficial properties [54,55,56]. Thus, MGH forms a promising therapy to replace antibiotics and povidone-iodine. From a practical and economical point of view, there are also other advantages. MGH only needs to be applied once daily, while povidone-iodine needs to be applied twice daily. Faster wound healing will not only result in fewer dressing changes but also demands less wound care and will improve the quality of life of patients in an important stage of their lives. No pain or discomfort was experienced by patients given the MGH treatment. Previously, even in preterm neonatal wound care, MGH application was considered safe and effective [57].

Although the presented research is a prospective cohort study and not a randomized controlled trial, there are several advantages of this study design. The relatively large sample size over an 11-month period and high participation rate (>76.7%) among all CS patients within this one hospital support the acceptability and appropriateness of the treatments in this specific target audience (real-world observation). Moreover, the study groups are well-matched, as indicated by the similarity in the baseline characteristics for both groups, subsequently limiting the attribution of confounders and improving internal validity. Since the group sizes were quite large, we were able to investigate protective and predisposing factors among the patient population and subsequently evaluate whether the complications impacted wound healing progression. Another strength of this study is that this study used an MGH formulation (L-Mesitran) that adheres to strict standards that ensure the safety, quality, and efficacy of the product. This is in contrast to most previous studies in which raw honey was used. A shortcoming may be the lack of randomization, blinding, or a placebo control group. However, the question arises as to whether blinding is possible and whether a proper placebo exists, considering a sugar solution with water may already result in a positive outcome by creating a moist wound environment, and the odor of honey can not be mimicked. Additionally, treatment regimens may differ; for example, the twice daily application of povidone-iodine would already reveal the study group. Future studies to substantiate these findings should preferably be large and multi-centered (double-)blinded RCTs.

## 4. Materials and Methods

### 4.1. Study Population, and (Non-)Inclusion and Exclusion Criteria

A prospective cohort study was performed in the Obstetrics and Gynecology Department at the Gabriel Touré University Hospital Center in Bamako, Mali. The study population consisted of patients admitted to the maternity wards to undergo a CS during the study period. The sample size was calculated based on the frequency of surgical site infections in the gynecology and obstetrics department of the Gabriel Touré University Hospital Center in 2017, which was 56% [58]. The sample size per group was calculated according to Daniel Schwartz’s formula [59] (N = (1.96)^2^ × 0.56 × 0.44/(0.05)^2^ = 378.63). The minimum sample size was thus 379 patients per group. In our study, a total of 766 patients were investigated, and divided into two equal groups of 383 patients.

**Figure 3:**
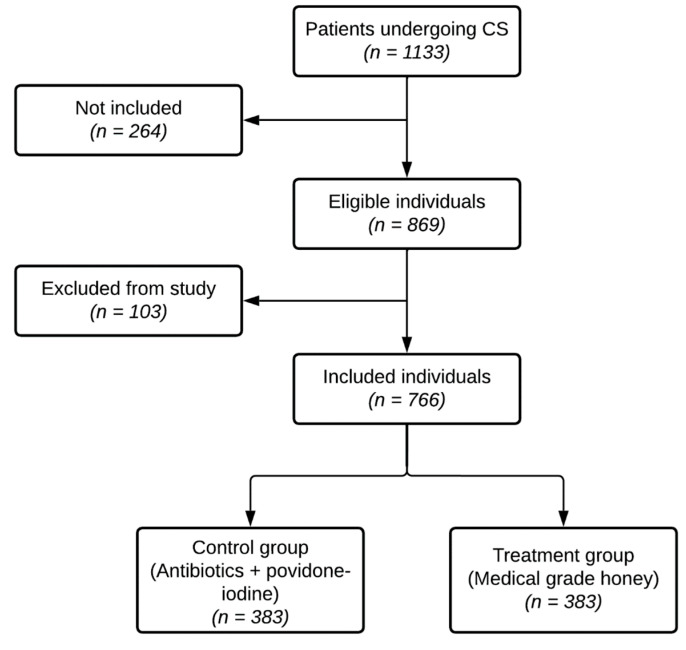
Flow diagram for inclusion and exclusion of patients.

The study started on 15 January 2020, and stopped after roughly 11 months on December 12, 2020, when all 766 patients were included in the study. In this period, 1133 patients underwent a CS (Figure 3). From this total population, 264 were not included in the study, because they did not agree to participate in the study or the CSs were performed in another clinic (non-inclusion). Patients who underwent a clean or clean-contaminated CS (Altmeier class I and II [60]) within the Obstetrics and Gynecology Department of the Gabriel Touré University Hospital Center and who consented to participate in the study (n = 869) were included (inclusion). Patients who did not follow the study protocol, or did not continue treatment within the Obstetrics and Gynecology Department of the Gabriel Touré University Hospital Center and all patients who died in the immediate postpartum period were excluded (n = 103)(exclusion). In total, 766 patients were included in the study and divided into either the treatment or control group (Figure 3).

The baseline characteristics (age, gravidity, parity, body mass index (BMI in kg/m^2^), education level, and occupation) from this patient population were recorded using a questionnaire (Table 1, Annex S1). The baseline characteristics were comparable between the two study groups. Additionally, the indication to perform a CS was comparable between the two groups (Table S1 [61]).

### 4.2. Ethical Statement

All subjects gave their informed consent for inclusion before they participated in the study. The study was conducted in accordance with the Declaration of Helsinki. Participation in the study was voluntary and the privacy of the participants has been maintained.

### 4.3. Treatment Protocol and Outcome Measures

The surgical site was prepared by applying a 10% povidone-iodine solution (Betadine dermal), which was subsequently left to dry for two to three minutes. Next, the CS procedure was performed, and the wound was sutured using a simple interrupted, subcuticular, or vertical mattress suture technique. The patients were advised not to wet the wounds until they had healed.

The postoperative wound care protocol was different for the two study groups. In the control group (antibiotics and povidone-iodine), the sutured wound was cleaned with 10% povidone-iodine applied using a soaked compress. The wound was then covered with a dry compress fixated using adhesive tape. Povidone-iodine was re-applied during the wound dressing changes twice per day according to the instructions for use. Treatment with antibiotics (amoxicillin) was started on day 1 post-operation and ended on day 5 if there were no complications. Otherwise, treatment was prolonged.

In the treatment (MGH) group, the cleaning of the wound was performed using saline solution, and then, the wound was dried using a dry compress. A thin layer of L-Mesitran Soft was applied to the primarily closed CS wound, which was subsequently covered with a compress fixated with adhesive tape. L-Mesitran Soft is a wound care formulation containing 40% MGH, and is enriched with other ingredients, including vitamins C and E, lanolin, PEG4000, and propylene glycol (www.mesitran.com, accessed on 6 December 2022; manufacturer: Theo manufacturing BV, Maastricht, the Netherlands). The honey used in L-Mesitran can be of different floral and geographical origin but always complies with strict MGH criteria to guarantee safety, quality, and efficacy [22]. Hence, MGH is free of pesticides, herbicides, heavy metals, antibiotics, and endospores/micro-organisms (due to gamma irradiation) and adheres to certain physicochemical characteristics [30]. The other ingredients enhance the antimicrobial activity and improve the applicability [54,55,56]. The wound dressings and MGH were refreshed once a day according to the instructions for use. No antibiotic or antiseptic treatment was administered.

The wounds were regularly monitored (on days 4, 7, 11, 15, and 30 post-operation), and in dialogue with the patient and using the questionnaire (Annex S1), wound healing was assessed (progression and dehiscence) and signs of inflammation and infection were examined (pain at the surgical site, fever (≥38.5 °C), superficial or deep pus discharge, crust formation, presence of wound exudate, peritonitis, endometritis, and wound bleeding). Postoperative complications were considered to be present when the patients experienced pain at the surgical site and had at least one other complication. The condition to be classified as having SSI was the presence of pus discharge and having pain at the surgical site. Half of the sutures were removed on day 7 and total ablation followed on day 11 according to the evolution of the wound, under the condition that there were no complications. The effects of different patient-independent factors on the complication risk were evaluated, including admission to hospital (own initiative or referral), the duration of hospitalization, the presence of membranes during CS, the delay between the rupture of the membranes and the performance of the CS, the form of anesthesia (general, spinal, or epidural), the duration of the CS procedure, and the suture technique.

### 4.4. Statistical Analysis

Data entry and analysis were performed using SPSS (IBM SPSS Statistics for Windows, version 22.0. IBM Corp., Armonk, NY, USA). The correlations between variables were analyzed using the Pearson’s test and Yates’ continuity correction. The Fisher–Yates exact test was used to determine the significance between the two groups. The reference values to determine the relative risks (RRs) were chosen within the control group to enable better interpretation of the RRs between the subgroups. Results were considered significantly different at *p* < 0.05.

## 5. Conclusions

The application of MGH considerably reduces the healing time of post-operative CS wounds. Moreover, MGH is as effective as antibiotics combined with povidone-iodine in preventing infections. Interestingly, due to the broad-spectrum antimicrobial activity of MGH by multiple mechanisms, there is no risk of developing antibiotic resistance to MGH, which is a huge problem in developing countries. The application of MGH is easy and safe and has practical benefits. MGH is cost-effective for the treatment of CS wounds and is a valuable and potent alternative to conventional treatments, such as antibiotics and topical povidone-iodine.

## Figures and Tables

**Table 1:** Baseline characteristics of the study population (383 patients per group). Patient characteristics are divided into subgroups and presented separately for both study groups.

Characteristic	Subgroup	Control Group (Antibiotics + Povidone-Iodine) n (%)	Treatment Group (MGH) n (%)
**Age**	≤19	69 (18.0)	78 (20.4)
	20–34	239 (62.4)	236 (61.6)
	≥35	75 (19.6)	69 (18.0)
**Gravidity**	Primigravida (1)	105 (27.4)	102 (26.6)
	Paucigravida (2 or 3)	114 (29.8)	124 (32.4)
	Multigravida (4 or 5)	88 (23.0)	93 (24.3)
	Grand multigravida (>5)	76 (19.8)	64 (16.7)
**Parity**	Nullipara (0)	112 (29.2)	106 (27.7)
	Primipara (1)	65 (17.0)	66 (17.2)
	Paucipara (2 or 3)	111 (29.0)	130 (33.9)
	Multipara (4 or 5)	76 (19.8)	47 (12.3)
	Grand multipara (>5)	19 (5.0)	34 (8.9)
**BMI**	≤18.5	5 (1.3)	6 (1.6)
	18.5–25	163 (42.5)	164 (42.8)
	25–30	142 (37.1)	131 (34.2)
	30–35	48 (12.5)	51 (13.3)
	35–40	19 (5.0)	21 (5.5)
	>40	6 (1.6)	10 (2.6)
**Education**	Primary	100 (26.1)	78 (20.4)
**level**	Secondary	68 (17.8)	79 (20.6)
	Tertiary	20 (5.2)	24 (6.3)
	No education	188 (49.1)	187 (48.8)
	Religious	7 (1.8)	15 (3.9)
**Occupation**	Housewife	278 (72.6)	267 (69.7)
	Store employee	47 (12.3)	46 (12.0)
	Government	37 (9.7)	44 (11.5)
	Student	19 (5.0)	24 (6.3)
	Other	2 (0.5)	2 (0.5)

**Table 2:** Overview of the number and percentage of postoperative complications and surgical site infections following CS. Data are presented per group (control, treatment, or total).

		Control Group (Antibiotics + Povidone-Iodine) n (%)	Treatment Group (MGH) n (%)	Total n (%)
Postoperative	Yes	74 (19.3)	72 (18.8)	146 (19.1)
complications	No	309 (80.7)	311 (81.2)	620 (80.9)
	**Total**	**383 (100)**	**383 (100)**	**766 (100)**
Surgical site	Yes	61 (15.9)	55 (14.4)	116 (15.1)
infections	No	322 (84.1)	328 (85.6)	650 (84.9)
	**Total**	**383 (100)**	**383 (100)**	**766 (100)**

**Table 3:** Overview of the different types of complications that were experienced by the patients presented per group. Both the control and treatment groups consisted of 383 patients, with 74 and 72 patients considered to have postoperative complications, respectively. Subgroups were based on the presence or absence of postoperative complications. Experiencing pain was a prerequisite, and therefore, the total number of patients with complications and patients experiencing pain are similar. Multiple complications were possible per patient. * represents a significant difference (*p* < 0.05).

	Control Group (Antibiotics + Povidone-Iodine)			Treatment Group (MGH)			
	Complication		RR (IC 95%) within Group	Complication		RR (IC 95%) within Group	RR (IC 95%) between Groups
Type of Complication	Yes n (%)	No n (%)		Yes n (%)	No n (%)		
Pain at the surgical site	74 (19.3)	309 (80.7)	-	72 (18.8)	311 (81.2)	-	0.97 [0.73–1.30]
Superficial pus discharge	31 (41.9)	43 (58.1)	3.88 * [1.91–7.86]	49 (68.1)	23 (31.9)	6.30 * [3.21–12.34]	1.62 * [1.19–2.22]
Deep pus discharge	30 (40.5)	44 (59.5)	3.75 * [1.84–7.63]	6 (8.3)	66 (91.7)	0.77 [0.28–2.11]	0.21 * [0.09–0.46]
Crust formation	8 (10.8)	66 (89.2)	Reference	0 (0)	72 (100)	0.06 [0.00–1.00]	0.06 [0.00–1.03]
Presence of wound exudate	0 (0)	74 (100)	0.06 [0.00–1.00]	7 (9.7)	65 (90.3)	0.90 [0.34–2.35]	15.41 [0.90–264.98]
Peritonitis	2 (2.7)	72 (97.3)	0.25 [0. 05–1.14]	1 (1.4)	71 (98.6)	0.13 [0.02–1.00]	0.51 [0.048–5.54]
Endometritis	0 (0)	74 (100)	0.06 [0.00–1.00]	1 (1.4)	71 (98.6)	0.13 [0.02–1.00]	3.08 [0.13–74.44]
Wound bleeding	5 (6.8)	69 (93.2)	0.63 [0.21–1.82]	13 (18.1)	59 (81.9)	1.67 [0.74–3.79]	2.67 * [1.00–7.11]

**Table 4:** Overview of patients who developed a fever within 30 days after the CS procedure, presented per study group. Both the control and treatment groups consisted of 383 patients, with 74 and 72 patients considered to have postoperative complications, respectively. Subgroups were based on the presence or absence of postoperative complications. * represents a significant difference (*p* < 0.05).

		Control Group (Antibiotics + Povidone-Iodine)			Treatment Group (MGH)		
		Complication		RR (IC 95%) within Group	Complication		RR (IC 95%) within Group
		Yes (%)	No (%)		Yes (%)	No (%)	
Fever between Day 0–30	Yes	5 (71.4)	2 (28.6)	10.44 * [2.07–52.76]	1 (33.3)	2 (66.7)	2.16 [0.20–23.49]
	No	69 (18.4)	307 (81.6)		71 (18.7)	309 (81.3)	

**Table 5:** The distribution of complications over different baseline characteristics per study group. Both the control and treatment groups consisted of 383 patients, with 74 and 72 patients considered to have postoperative complications, respectively. Subgroups were based on the presence or absence of postoperative complications. * represents a significant difference (*p* < 0.05).

		Control Group (Antibiotics + Povidone-Iodine)			Treatment Group (MGH)			
		Complication		RR (IC 95%) within Group	Complication		RR (IC 95%) within Group	RR (IC 95%) between Groups
		Yes (%)	No (%)		Yes (%)	No (%)		
**Age (years)**	≤19	13 (18.8)	56 (81.2)	0.87 [0.50–1.49]	8 (10.3)	70 (89.7)	0.47 * [0.23–0.95]	0.54 [0.24–1.23]
	20–34	52 (21.8)	187 (78.2)	Reference	53 (16.5)	183 (77.5)	1.03 [0.74–1.45]	1.03 [0.74–1.45]
	≥ 35	9 (12.0)	66 (88.0)	0.55 [0.29–1.07]	11 (15.9)	58 (84.1)	0.73 [0.41–1.33]	1.33 [0.59–3.01]
**Gravidity**	Primigravida (1)	27 (25.7)	78 (74.3)	1.40 [0.84–2.31]	17 (16.7)	85 (83.3)	0.90 [0.51–1.62]	0.65 [0.38–1.11]
	Paucigravida (2 or 3)	21 (18.4)	93 (81.6)	Reference	20 (16.1)	104 (83.9)	0.88 [0.50–1.53]	0.88 [0.50–1.53]
	Multigravida (4 or 5)	15 (17.0)	73 (83.0)	0.93 [0.51–1.69]	18 (19.4)	75 (80.6)	1.05 [0.60–1.85]	1.14 [0.61–2.11]
	Grand multigravida (>5)	11 (14.5)	65 (85.5)	0.79 [0.40–1.53]	17 (26.6)	47 (73.4)	1.44 [0.82–2.53]	1.84 [0.93–3.63]
**Parity**	Nullipara (0)	27 (24.1)	85 (75.9)	Reference	17 (16.0)	89 (84.0)	0.67 [0.39–1.15]	0.67 [0.39–1.15]
	Primipara (1)	11 (16.9)	54 (83.1)	0.70 [0.37–1.32]	10 (15.2)	56 (84.8)	0.63 [0.33–1.21]	0.90 [0.41–1.96]
	Paucipara (2 or 3)	26 (23.4)	85 (76.6)	0.97 [0.61–1.56]	26 (20.0)	104 (80.0)	0.83 [0.52–1.33]	0.85 [0.41–1.96]
	Multipara (4 or 5)	9 (11.8)	67 (88.2)	0.49 [0.24–0.99]	11 (23.4)	36 (76.6)	0.97 [0.53–1.79]	1.98 [0.89–4.41]
	Grand multipara (>5)	1 (5.3)	18 (94.7)	0.21 [0.03–1.51]	8 (23.5)	26 (76.5)	0.98 [0.49–1.95]	4.47 [0.60–33.09]
**BMI (kg/m^2^)**	≤18.5	3 (60.0)	2 (40.0)	3.15 * [1.44–6.90]	1 (16.7)	5 (83.3)	0.88 [0.14–5.39]	0.28 [0.04–1.91]
	18.5–25	31 (19.0)	132 (81.0)	Reference	21 (12.8)	143 (87.2)	0.67 [0.40–1.12]	0.67 [0.40–1.12
	25–30	25 (17.6)	117 (82.4)	0.93 [0.57–1.49]	33 (25.2)	98 (74.8)	1.32 [0.86–2.04]	1.43 [0.90–2.27]
	30–35	9 (18.8)	39 (81.2)	0.99 [0.51–1.92]	11 (21.6)	40 (78.4)	1.13 [0.62–2.09]	1.15 [0.52–2.53]
	35–40	3 (15.8)	16 (84.2)	0.83 [0.28–2.46]	3 (14.3)	18 (85.7)	0.75 [0.25–2.24]	0.90 [0.21–3.96]
	> 40	3 (50.0)	3 (50.0)	2.63* [1.11–6.22]	3 (30.0)	7 (70.0)	1.58 [0.58–4.28]	0.60 [0.17–2.07]
**Education level**	Primary	19 (19.0)	81 (81.0)	1.02 [0.62–1.69]	17 (21.8)	61 (78.2)	1.17 [0.70–1.96]	1.15 [0.64–2.06]
	Secondary	19 (27.9)	49 (72.1)	1.50 [0.92–2.44]	15 (19.0)	64 (81.0)	1.02 [0.59–1.76]	0.68 [0.38–1.23]
	Tertiary	1 (5.0)	19 (95.0)	0.27 [0.04–1.86]	3 (12.5)	21 (87.5)	0.76 [0.22–2.02]	2.50 [0.28–22.21]
	No education	35 (18.6)	153 (81.4)	Reference	35 (18.7)	152 (81.3)	1.01 [0.66–1.53]	1.01 [0.66–1.53]
	Religious	0 (0)	7 (100.0)	0.33 [0.02–4.95]	2 (13.3)	13 (86.7)	0.72 [0.19–2.69]	2.50 [0.14–46.14]
**Occupation**	Housewife	55 (19.8)	223 (80.2)	Reference	52 (19.5)	215 (80.5)	0.98 [0.7–1.38]	0.98 [0.70–1.38]
	Store employee	9 (19.1)	38 (80.9)	0.97 [0.51–1.82]	11 (23.9)	35 (76.1)	1.21 [0.69–2.13]	1.25 [0.57–2.73]
	Government	4 (10.8)	33 (89.2)	0.55 [0.21–1.42]	7 (15.9)	37 (84.1)	0.80 [0.39–1.65]	1.47 [0.47–4.64]
	Student	6 (31.6)	13 (68.4)	1.60 [0.79–3.22]	1 (4.2)	23 (95.8)	0.21 [0.03–1.46]	0.13 [0.02–1.004]
	Other	0 (0)	2 (100)	0.84 [0.07–10.64]	1 (50)	1 (50)	2.53 [0.62–10.31]	3.00 [0.19–47.97]

**Table 6:** Overview of distribution of complications by non-baseline-related factors in both groups. Both the control and treatment groups consisted of 383 patients, with 74 and 72 patients considered to have postoperative complications, respectively. Subgroups were based on the presence or absence of postoperative complications. * represents a significant difference (*p* < 0.05).

		Control Group (Antibiotics + Povidone-Iodine)			Treatment Group (MGH)			
		Complication		RR (IC 95%) within Group	Complication		RR (IC 95%) within Group	RR (IC 95%) between Groups
		Yes (%)	No (%)		Yes (%)	No (%)		
**Admission to the hospital**	On own initiative	13 (14.0)	80 (86.0)	0.66 [0.38–1.15]	17 (22.1)	60 (77.9)	1.05 [0.65–1.69]	1.58 [0.82–3.04]
	Referral	61 (21.0)	229 (79.0)	Reference	55 (18.0)	251 (82.0)	0.85 [0.62–1.19]	0.85 [0.62–1.18]
**Duration of hospitalization (in days)**	≤5	58 (21.1)	217 (78.9)	Reference	47 (17.0)	229 (83.0)	0.81 [0.57–1.14]	0.81 [0.57–1.14]
	5 d–10 d	11 (11.1)	88 (88.9)	0.53 * [0.29–0.96]	24 (23.5)	78 (76.5)	1.12 [0.73–1.69]	2.12 * [1.10–4.09]
	10 d–15 d	5 (55.6)	4 (44.4)	2.63 * [1.41–4.93]	1 (20.0)	4 (80.0)	0.95 [0.16–5.56]	0.36 [0.06–2.28]
**Presence of membranes during CS**	Intact	58 (19.4)	241 (80.6)	Reference	61 (19.4)	254 (80.6)	1.00 [0.72–1.38]	1.00 [0.72–1.38]
	Not intact	16 (19.0)	68 (81.0)	0.98 [0.60–1.62]	11 (16.2)	57 (83.8)	0.83 [0.46–1.50]	0.85 [0.42–1.71]
**Delay between rupture of membranes (in hours)**	≤24 h	15 (21.4)	55 (78.6)	Reference	11 (16.4)	56 (83.6)	0.77 [0.38–1.55]	0.77 [0.38–1.55]
	24 h–72 h	1 (16.7)	5 (83.3)	0.78 [0.12–4.92]	0 (0)	1 (100.0)	1.15 [0.10–13.15]	1.17 [0.07–18.96]
	72 h–100 h	0 (0)	5 (100.0)	0.38 [0.03–5.62]	0 (0)	0 (0)	2.29 [0.31–17.07]	6.00 [0.22–162.54]
	≥120 h	0 (0)	1 (100.0)	1.15 [0.10–13.15]	0 (0)	0 (0)	2.29 [0.31–17.07]	2.00 [0.09–44.35]
**Anesthesia**	General	37 (50)	100 (32.4)	1.78 * [1.19–2.67]	32 (25.0)	96 (75.0)	1.65 * [1.08–2.51]	0.93 [0.62–1.39]
	Spinal	37 (17.0)	207 (83.0)	Reference	39 (15.6)	211 (84.4)	1.03 [0.68–1.56]	1.03 [0.68–1.56]
	Epidural	0 (0.0)	2 (100.0)	1.09 [0.09–13.91]	1 (20.0)	4 (80.0)	1.32 [0.22–7.81]	1.50 [0.08–26.86]
**Duration of CS**	15–30 min	15 (16.7)	75 (83.3)	0.91 [0.53–1.57]	12 (12.5)	84 (87.5)	0.68 [0.37–1.24]	0.75 [0.37–1.51]
	30–45 min	39 (18.3)	174 (81.7)	Reference	43 (19.3)	180 (80.7)	1.05 [0.71–1.56]	1.05 [0.71–1.56]
	45–60 min	15 (23.4)	49 (76.6)	1.28 [0.76–2.17]	16 (35.6)	29 (64.4)	1.94 * [1.20–3.15]	1.52 [0.84–2.74]
	≥60 min	5 (31.3)	11 (68.7)	1.71 [0.78–3.72]	1 (5.3)	18 (94.7)	0.29 [0.4–1.98]	0.17 [0.02–1.30]
**Suture technique**	Simple interrupted	70 (21.5)	256 (78.5)	Reference	60 (20.2)	237 (79.8)	0.94 [0.69–1.28]	0.94 [0.69–1.28]
	Vertical mattress	0 (0)	24 (100)	0.09 [0.01–1.45]	2 (12.5)	14 (87.5)	0.58 [0.16–2.16]	7.35 [0.38–143.79]
	Subcuticular	4 (12.1)	29 (87.9)	0.56 [0.22–1.45]	10 (14.3)	60 (85.7)	0.66 [0.36–1.22]	1.18 [0.40–3.48]

**Table 7:** Overview of wound healing time per study group. Both the control and treatment groups consisted of 383 patients, with 74 and 72 patients considered to have postoperative complications, respectively. Subgroups were based on the presence or absence of postoperative complications. * represents a significant difference (*p* < 0.05).

Duration of Wound Healing (Days)	Control Group (Antibiotics + Povidone-Iodine)			Treatment Group (MGH)		
	Complication		RR (IC 95%) within Group	Complication		RR (IC 95%) within Group
	Yes n (%)	No n (%)		Yes n (%)	No n (%)	
**7–14**	0 (0)	26 (100.0)	0.24 [0.01–4.11]	2 (2.6)	76 (97.4)	0.35 [0.07–1.71]
**14–21**	6 (7.2)	77 (92.8)	Reference	15 (8.9)	153 (91.1)	1.23 [0.50–3.07]
**21–28**	27 (14.9)	154 (85.1)	2.06 [0.88–4.81]	28 (29.8)	66 (70.2)	4.12 * [1.80–9.46]
**28–35**	12 (26.1)	34 (73.9)	3.61 * [1.45–8.98]	11 (42.3)	15 (57.7)	5.85 * [2.40–14.28]
**35–42**	12 (40.0)	18 (60.0)	5.53 * [2.28–13.43]	10 (100.0)	0 (0)	13.83 * [6.40–29.90]
**42–49**	9 (100.0)	0 (0)	13.83 * [6.40–29.90]	2 (66.7)	1 (33.3)	9.22 * [3.04–28.01]
**49–56**	8 (100.0)	0 (0)	13.83 * [6.40–29.90]	4 (100.0)	0 (0)	13.83 * [6.40–29.90]

**Table 8:** Overview of wound healing time per suture technique (simple interrupted, vertical mattress, and subcuticular). The percentages represent the distribution over the two study groups for the specific suture technique.

Duration of Wound Healing in Days	Control Group (Antibiotics + Povidone-Iodine)			Treatment Group (MGH)		
	Simple Interrupted Suture n (%)	Vertical Mattress Suture n (%)	Subcuticular Suture n (%)	Simple Interrupted Suture n (%)	Vertical Mattress Suture n (%)	Subcuticular Suture n (%)
**7–14**	15 (45.5)	3 (42.9)	8 (12.5)	18 (54.5)	4 (57.1)	56 (87.5)
**14–21**	47 (24.1)	12 (50.0)	24 (75.0)	148 (75.9)	12 (50.0)	8 (25.0)
**21–28**	172 (66.2)	9 (100.0)	0 (0)	88 (33.8)	0 (0)	6 (100.0)
**28–35**	45 (63.4)	0 (0)	1 (100.0)	26 (36.6)	0 (0)	0 (0)
**35–42**	30 (75.0)	0 (0)	0 (0)	10 (25.0)	0 (0)	0 (0)
**42–49**	9 (75.0)	0 (0)	0 (0)	3 (25.0)	0 (0)	0 (0)
**49–56**	8 (66.7)	0 (0)	0 (0)	4 (33.0)	0 (0)	0 (0)
**Total**	**326 (85.1)**	**24 (6.3)**	**33 (8.6)**	**297 (77.5)**	**16 (4.2)**	**70 (18.3)**

## Data Availability

The data that support the findings of this study are available from the corresponding author upon reasonable request. All data relevant to the study are included in the article.

## Supplementary Materials

The following supporting information can be downloaded at: https://www.mdpi.com/article/10.3390/antibiotics12010092/s1, Table S1: Indication to perform a CS per study group; Annex S1: questionnaire.
