# Supplementary material for: Medical-Grade Honey Enhances the Healing of Caesarean Section Wounds and Is Similarly Effective to Antibiotics Combined with Povidone-Iodine in the Prevention of Infections—A Prospective Cohort Study
Source: Antibiotics (Basel). 2023 Jan 5;12(1):92. doi: 10.3390/antibiotics12010092 (PMC9855001; doi:10.3390/antibiotics12010092)
Supplement: Supplementary file 1 [file antibiotics-12-00092-s001.zip › antibiotics-2114040-supplementary.pdf]

Table S1: Indication to perform a CS per study group. There were 383 patients per group, multiple indications per group are possible. The presented percentages represent the relative group of patients with a certain indication within the specified group[61].

Indication	Control group (Antibiotics + povidone-iodine) n, (%)	Treatment group (MGH) n, (%)	Total n, (%)
Pre-eclampsia	88, (23)	88, (23)	176, (23)
Asherman's Syndrome (AS)/ Intrauterine adhesions	74, (19.3)	79, (20.6)	153, (20)
Retroplacental hematoma, SHER Class II/IIIa [61]	74, (19.3)	73, (19.1)	147, (19.2)
Eclampsia	54, (14.1)	64, (16.7)	118, (15.4)
Acute fetal distress	46, (12.0)	44, (11.6)	90, (11.7)
Narrow pelvis	40, (10.4)	37, (9.7)	77, (10.1)
Abnormal fetal presentation	41, (10.7)	32, (8.4)	73, (9.5)
Asherman's Syndrome (AS) + narrow pelvis	30, (7.8)	24, (6.3)	54, (7.1)
Chronic fetal distress	16, (4.2)	16, (4.2)	32, (4.2)
Premature rupture of membranes	11, (2.9)	15, (3.9)	26, (3.4)
Placenta previa hemorrhage	7, (1.8)	15, (3.9)	22, (2.9)
Fetal macrosomia	6, (1.6)	6, (1.6)	12, (1.6)
Short birth interval + AS	5, (1.3)	6, (1.6)	11, (1.4)
Heart disease	5, (1.3)	1, (0.3)	6, (0.8)
Severe arterial hypertension	3, (0.8)	1, (0.3)	4, (0.5)
Umbilical cord prolapse	0, (0)	4, (1)	4, (0.5)
Myoma previa	1, (0.3)	3, (0.8)	4, (0.5)
Hydrocephalus	1, (0.3)	3, (0.8)	4, (0.5)
Acute lung edema	3, (0.8)	0, (0)	3, (0.4)
Nephropathy	3, (0.8)	0, (0)	3, (0.4)
AS + severe arterial hypertension	3, (0.8)	0, (0)	3, (0.4)
AS + post-term	0, (0)	3, (0.8)	3, (0.4)
Sickle cell anemia	3, (0.8)	0, (0)	3, (0.4)
Abnormal fetal heart rate	3, (0.8)	0, (0)	3, (0.4)
Other	5, (1.3)	3, (0.8)	8, (1)

Annex S1: Questionnaire

Q1). Date of entry: ……../…………/…….
Q2). Study number: ……………….
Q3). Surname(s) and first name(s): ……………………………………..
Q4). Tel: ……………………………………………..
Q5). Age: …………. years
Q6). Height: ………… cm
Q7). Weight: ………….. kg
Q8: BMI: ………………..kg/ m2
Q9). Place of residence: ………………. 1. Bamako 2. Outside of Bamako
Q10). Ethnic group: ………………… 1. Bambara 2. Peulh 3. Sonrhaï 4. Malinké 5. Dogon 6. Other
Q11). Marital status: ………….. 1. Married 2. Single 3. Divorced 4. Widowed
Q12). Profession: …………….. 1. Housewife 2. Store employee 3. Government 4. Student 5. Other
Q13). Level of education: ………………. 1. Primary 2. Secondary 3. Tertiary 4. No education 5. Religious
Q14). Mode of admission: …………….. 1. On own initiative 2. Referral
Q15). Reason for exclusion: ……………………………..
Q16). Medical background
Family history: …………………. 1. Yes 2. No
o Arterial hypertension
o Diabetes
o Heart disease
o Asthma
o Epilepsy
o Sickle cell disease
o Other: ……………………………..

Medical history: …………………. 1. Yes 2. No
o Arterial hypertension
o Diabetes
o Heart disease
o Asthma
o HIV
o Epilepsy
o Sickle cell disease
o Eclampsia
o Hepatitis B
o Other: ……………………………..

Surgical history: …………………. 1. Yes 2. No
o Caesarean section
o Uterine rupture

o Salpingectomy
o Other(s): ……………………………..

Obstetric history: Gestation ……….. Parity ……... Alive …….… Deceased ……… Aborted………..

Q17). Pregnancy age: …………………… years
Q18). Number of prenatal consultations
Q19). Indication of Caesarean section
o Pre-eclampsia
o Asherman's Syndrome (AS)/ Intrauterine adhesions
o Retroplacental hematoma, SHER Class II/IIIa
o Eclampsia
o Acute fetal distress
o Narrow pelvis
o Abnormal fetal presentation
o Asherman's Syndrome (AS) + narrow pelvis
o Chronic fetal distress
o Premature rupture of membranes
o Placenta previa hemorrhage
o Fetal macrosomia
o Short birth interval + AS
o Heart disease
o Severe arterial hypertension
o Umbilical cord prolapse
o Myoma previa
o Hydrocephalus
o Acute lung edema
o Nephropathy
o AS + severe arterial hypertension
o AS + post-term
o Sickle cell anemia
o Abnormal fetal heart rate
o Other(s): ……………………………..

Q20). Type of anesthesia /…………../ 1. General 2. Spinal anesthesia 3. Epidural
Q21). Presence of membrane during CS /…………../ 1. Intact 2. Not intact
If not intact, time between rupture and Caesarean section ……… hours
Q22). Duration of Caesarean section ……….. minutes
Q23). Suture type /…………../ 1. Simple interrupted 2. Vertical mattress 3. Subcuticular
Q24). Postoperative medical treatment /…………../ 1. Antibiotic therapy + local antiseptics 2. Medical grade honey
Q25). Duration of hospitalization ………….. days
Q26). Duration of treatment ………….. days

Q27). Duration of healing ………….. days
Q28). Postoperative complications /…………../ 1. Yes 2. No
If yes, type of complications:
o Pain at the surgical site
o Superficial pus discharge
o Deep pus discharge
o Curst formation
o Presence of wound exudate
o Peritonitis
o Endometritis
o Wound bleeding
o Other(s): ……………………………..

Q29). Fever /…………../ 1. Yes 2. No
If yes, temperature ………… °C
Q30). Date of onset of fever postoperatively /…………../ 1. Day 1 2. Day 2 3. Day 3 4. Day 4 5. Other: …. Day
Q31). Circumstances of onset of fever /…………../ 1. Pus discharge 2. Bleeding 3. Redness/ heat 4. Other:…….
Q32). Pain /…………../ 1. Yes 2. No
If yes, time of appearance ………… hours
Q33). Postoperative hemoglobin level …………… g/dl
Q34). Time to onset of complications ……………. Days

Q35). Monitoring items
Other ………………
Day 7
o Pain
o Fever
o Superficial pus discharge
o Deep pus discharge
o Wound bleeding
o Other(s): ……………………………..

Day 11
o Pain
o Fever
o Superficial pus discharge
o Deep pus discharge
o Wound bleeding
o Other(s): ……………………………..

Day 15

o Pain
o Fever
o Superficial pus discharge
o Deep pus discharge
o Wound bleeding
o Other(s): ……………………………..

Day 30
o Pain
o Fever
o Superficial pus discharge
o Deep pus discharge
o Wound bleeding
o Other(s): ……………………………..

Q36). Death /…………../ 1. Yes 2. No
If yes, cause(s) ………………
Time of occurrence of death …………… days
